# Design and Operation of an Optically-Accessible Modular Reactor for Diagnostics of Thermal Thin Film Deposition Processes

**DOI:** 10.6028/jres.120.005

**Published:** 2015-04-07

**Authors:** W. A. Kimes, B. A. Sperling, J. E. Maslars

**Affiliations:** National Institute of Standards and Technology, Gaithersburg, MD 20899

**Keywords:** ALD, atomic layer deposition, chemical vapor deposition, CVD, diagnostics, in situ, optical cell, reactor

## Abstract

The design and operation of a simple, optically-accessible modular reactor for probing thermal thin film deposition processes, such as atomic layer deposition processes (ALD) and chemical vapor deposition (CVD), is described. This reactor has a nominal footprint of 225 cm^2^ and a mass of approximately 6.6 kg, making it small enough to conveniently function as a modular component of an optical train. The design is simple, making fabrication straightforward and relatively inexpensive. Reactor operation is characterized using two infrared absorption measurements to determine exhaust times for tetrakis(dimethylamino)titanium and water, proto-typical ALD precursors, in a pressure and flow regime commonly used for ALD.

## 1. Introduction

Atomic layer deposition (ALD) is a thin film deposition technique [1] increasingly used in the microelectronics industry [2,3] due to its ability to deposit uniform, conformal, pin-hole free thin films in high aspect ratio features with sub-nanometer thickness control. ALD is generally understood as an iterative deposition process which deposits one monolayer, or sub-monolayer, per iteration using a self-limiting surface reaction achieved by sequentially exposing a deposition surface to alternating gas-phase precursors. The details of the chemistries involved are not well understood for all precursors.

*In situ* optical diagnostics have been demonstrated as valuable tools for investigating numerous ALD chemistries [4]. An integral aspect of *in situ* diagnostics is reactor design, which must permit optical access without significant modification of the desired deposition chemistry under investigation. A variety of reactor chamber designs have been reported for ALD growth [3,5,6], each type presents different issues when designing diagnostic access. The focus of this report is on a perpendicular-flow, single-wafer thermal ALD reactor chamber design for microelectronics applications. This class of reactors is designed to temporally separate precursors and maximize wafer throughput by minimizing ALD cycle times through short gas residence times. Two reactor design characteristics important for achieving short gas residence times are 1) all reactor volumes must be efficiently swept by inert purge gases to minimize stagnant gas volumes which can result in precursor mixing; and 2) all reactor surfaces must be effectively heated to both minimize the presence of cold surfaces (on which species can condense and subsequently desorb over time) and to minimize hot spots (which may result in precursor decomposition). A reactor design that combines these two characteristics with optical access is not generally commercially available or easily realized using off-the-shelf vacuum components.

In past works we presented the design and characterization of a diagnostics-accessible ALD reactor [7], which provided good optical access [8,9] and was capable of depositing device quality oxides [10]. The complexity and size of that reactor limited the ease in which it could be integrated into an optical train, making it impractical for use with a wide range of optical measurements. Therefore, we designed a modular reactor to emulate the deposition characteristics and optical access of the previous design, yet be small and simple enough to be easily manufactured, ported and mounted on an optical table – thereby allowing it to act as a modular component in an optical train. Previous publications have demonstrated the capability of the reactor to deposit device quality oxides using ALD [11] and to operate in either an ALD or chemical vapor deposition configuration [12]. However, a detailed report on the design and operation of the reactor has not been published. The objective of this report is to present the design details for this modular diagnostic-accessible reactor and to provide insight into the operation of the reactor for ALD. Two infrared absorption measurements were used to demonstrate a reactor configuration for use with optical measurements and to characterize purge times for tetrakis(dimethylamino)titanium (TDMAT) and water in a typical pressure and flow regime for ALD. TDMAT and water were chosen because they are prototypical ALD precursors which, due to the large difference in mass and collision diameters, have significantly different mass diffusivity values.

## 2. Reactor Design

**Fig. 1:**
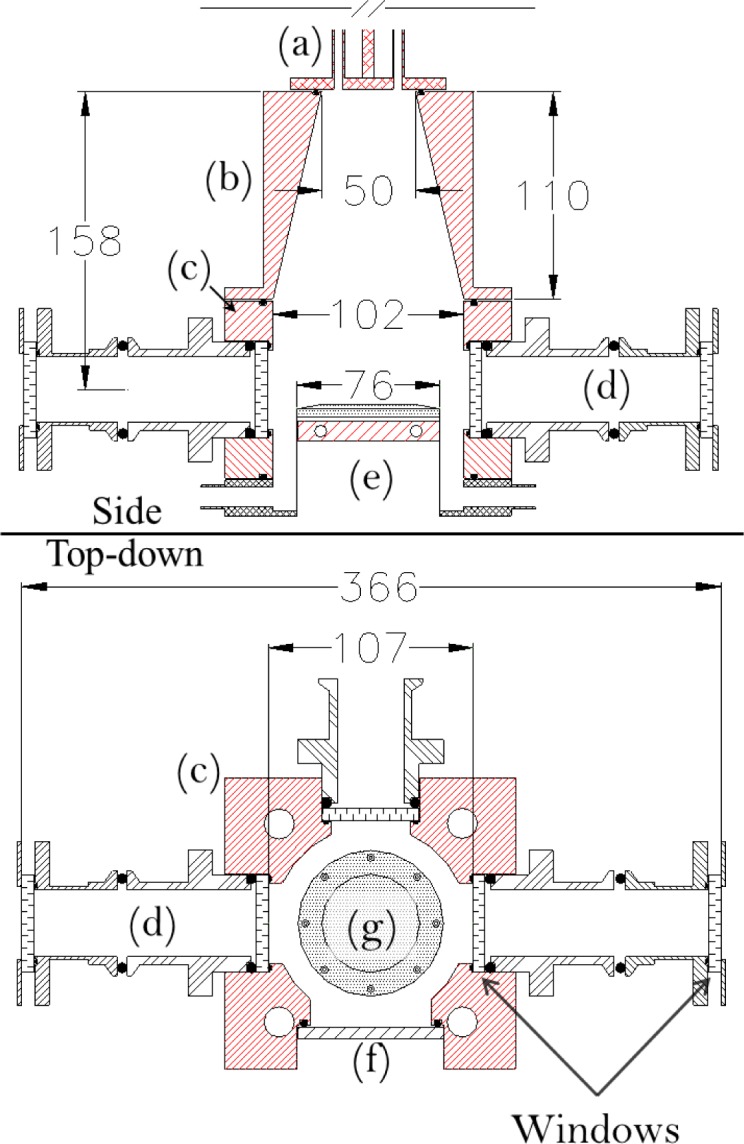
Cross sectional side view (top) and top-down view (bottom) of the reactor. Gas flow, in the cross sectional view, is from the top of the page towards the bottom of the page. Dimensions are in millimeters. The red portions indicate areas which are actively heated. (a) Injection lines; (b) expansion cone; (c) reactor body; (d) diagnostic ports with a double window configuration; (e) exhaust manifold; (f) loading port; (g) aluminum face plate located on wafer chuck.

Figure 1 shows a cross sectional side view and top down view of the modular reactor design. The reactor is formed primarily from three components, vacuum-sealed to one another using perfluoroelastomer O-rings: 1) an expansion cone, consisting of a cylinder with a conical cutout, 2) the reactor body, consisting of a simple flow tube with recessed diagnostic ports, and 3) the exhaust manifold with an integrated wafer chuck.

The expansion cone (see (b) Fig. 1) is a 112 mm outer diameter (OD), 110 mm long aluminum cylinder with a conical cutout which expands from a 50 mm inner diameter (ID) to 102 mm ID over the length of the cone. The expansion cone is heated using a wire wrap kapton heater.

The reactor body (see (c) Fig. 1) is an aluminum cuboid, 94 mm long with a 152 mm × 152 mm square cross section and a 102 mm diameter hole bored through the center. The reactor body has three diagnostic ports (see (d) Fig. 1), and one loading port (see (f) Fig. 1), all located on the same plane (47 mm from the top of the cell body). Each port is composed of a recessed well that is designed to seal a circular disk as close as possible to the ID of the center bore using an elastomer O-ring. The three diagnostic ports are designed to accommodate 50 mm diameter windows, providing a clear optical aperture of 42 mm and a nominal optical path length (between the interior window surfaces) of 107 mm. The loading port is designed to accommodate a 76 mm disk, providing a clear aperture of 64 mm. The reactor body is heated using four cartridge heaters embedded in the body.

The exhaust manifold (see (e) Fig. 1) is formed by combining an exhaust flange with a wafer chuck. The body of the exhaust flange is a 152 mm diameter, 19 mm thick stainless steel disk with a 102 mm diameter, 17 mm deep cylindrical recess. The recess acts to extend the length of the center bore in the reactor body, when the exhaust manifold is mounted to the reactor body. Gases are exhausted through four radially symmetric 13 mm OD, 10 mm ID tubes located on the wall of the recess. The wafer chuck is a 75.7 mm ID, 76.2 mm OD stainless steel cylinder welded to the bottom of the recess, such that it seals a 76.2 mm diameter through hole. This configuration allows the inside of the wafer chuck cylinder to be exposed to air while the outer surface is under vacuum. The wafer chuck is heated using a 75.5 mm diameter, 10 mm thick copper disk with embedded cartridge heaters attached to the inside of the wafer chuck, such that the heaters are exposed to air. A 76.2 mm diameter, 6 mm thick aluminum face place is bolted to the vacuum side of the wafer chuck, to act as a thermal diffuser. The body of the exhaust manifold is not actively heated.

## 3. Reactor Configuration for Optical Measurements

Gasses were introduced into the expansion cone using four symmetric ≈4.8 mm ID, ≈6.3 mm OD (quarter inch OD) tubes (see (a) Fig. 1). A constant flow of purified nitrogen gas was maintained at 75 mL/min (273.15 K and 101.3 kPa) in each of the four injection tubes using pressure-based mass flow controllers. Pressure in the reactor was passively maintained at approximately 133 Pa. Gas flow was directed using computer-controlled pneumatic diaphragm valves. TDMAT was injected into the reactor by redirecting the gas flow of one of the four injection tubes through a heated bubbler. Measurements for TDMAT were taken during a series of 50 consecutive iterations of 2 s nitrogen only, 2 s nitrogen with TDMAT, and 5 s nitrogen only. Water was injected into the reactor by introducing the vapor of a liquid water vessel through a heated needle valve into one of the three injection tubes not used for TDMAT, by means of a three-way valve. Injection transients for water were minimized by pumping on the water vessel through the needle valve 5 s immediately prior to each water injection. Measurements for water were taken during a series of 25 iterations of 6 s nitrogen only, 2.5 s nitrogen with water, and 15 s nitrogen only. The injection tube for TDMAT was heated to nominally 348 K. The remaining three injection tubes, the water needle valve, and the reactor body were heated to nominally 383 K.

The reactor was instrumented using a double window configuration (see (d) Fig. 1) to reduce noise attributed to beam steering resulting from turbulent mixing of the cool ambient with the heated air near the windows. A set of four nominally identical 50 mm diameter circular window wedges, with a 0.5° wedge angle, were used for each measurement; ZnSe and CaF_2_ windows for TDMAT and water measurements, respectively.

## 4. Precursor Concentration Measurements

The TDMAT concentration as a function of time was determined by measuring the absorbance of the TDMAT feature at ≈948 cm^−1^ using a broadband infrared source in conjunction with a nominal 10.5 µm center wavelength bandpass filter, as previously described [8]. Data presented is an average of 50 injections, normalized to the maximum absorbance value measured during the pulse.

The water concentration as a function of time was determined by monitoring the rovibrational transition of water at 7181.156 cm^−1^ using a distributive feed-back diode laser and a wavelength modulation detection scheme, as previously described [13]. Data presented is an average of 25 injections, normalized to the maximum signal level detected during the pulse.

## 5. Results and Discussion

**Fig. 2:**
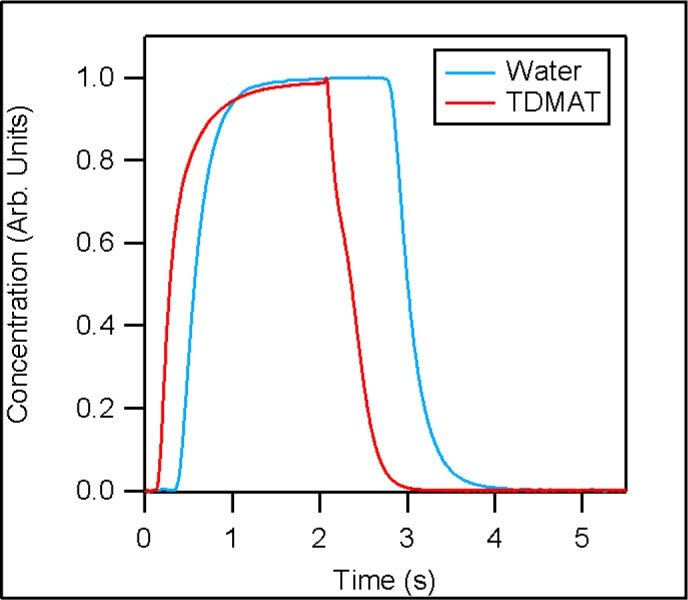
The normalized precursor concentration as a function of time for a 2 s TDMAT pulse and a 2.5 s water pulse.

Figure 2 depicts the normalized concentration profiles for a 2 s TDMAT pulse and a 2.5 s water pulse with respect to time. In this figure, time zero corresponds to the time when the valve connecting the precursor to an injection tube was signaled to open. While an in-depth analysis of the concentration profiles is beyond the scope of this paper, these profiles can be used to determine general guidelines for reactor purge times by examining the trailing edge of the each precursor pulse.

**Fig 3:**
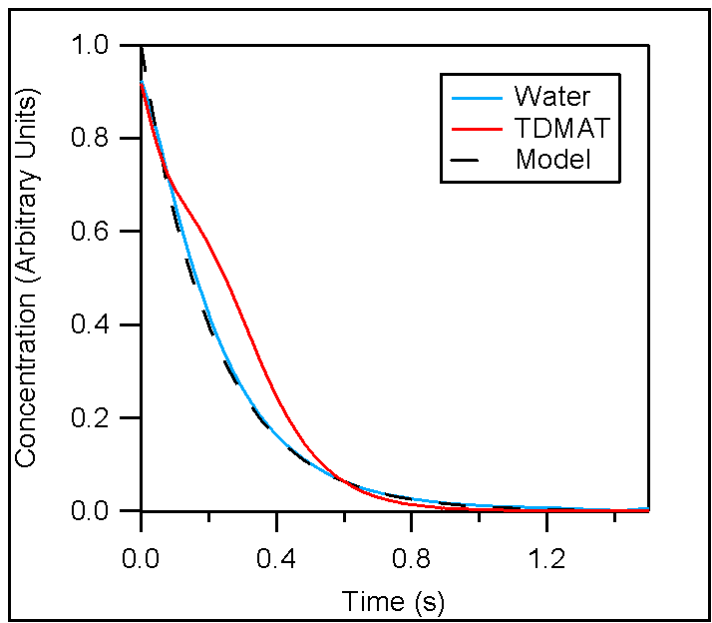
A comparison between the trailing edges of the normalized precursor concentration profiles and a simple exponential decay model.

Figure 3 depicts the trailing edge of both TDMAT and water concentration profiles compared against a simple exponential decay model. In this figure time zero corresponds to the beginning of each trailing edge. The beginning of the trailing edge is defined as the time when the valve connecting the precursor to an injection tube was signaled to close, plus the time required for the precursor to traverse the injection system. The traversal time was measured using the difference in time between the precursor valve opening and the time corresponding to the inflection point at the beginning of the pulse (see Fig. 2). Both trailing edge curves roughly approximate an exponential decay, suggesting the use of a simple dilution model is appropriate to approximate purge times. We compare the trailing edge against a simple model, described in Eq. (1), which assumes a uniform precursor distribution, a constant uniform temperature and that all changes to precursor concentration are the result of flows in and out of the reactor. The result is a precursor concentration (P(t)) curve described by an exponential decay, defined by an initial precursor concentration (P_0_ = 1) with an exponential decay constant equal to the ratio between volumetric flow rate of the purge gas (F = 5.33 L/s, at 373 K and 133 Pa) and the calculated reactor volume upstream of the face plate (V = 1.16 L):

$$P(t)=P_{0}e^{-t\frac{F}{V}}.$$ (1)

Agreement between the model and the measurement results for water is much better than that for TDMAT. This difference is likely the result of TDMAT being more efficiently entrained in the carrier gas flow due the smaller diffusion coefficient of TDMAT relative to water, resulting from the larger collision diameter and mass of TDMAT. In both cases agreement between the model and precursor concentration converge as the precursor concentrations approaches zero, suggesting the model is a useful tool for approximating purge times. For reactor pressures of 133 Pa and purge gas flow rates of 300 mL/min (at 273.15 K and 101.3 kPa) the model predicts a reduction of precursor concentrations by greater than a factor of 10^6^ after a 3.5 s purge. This purge time accounts for both reactor exhaust times and time required for each precursor to traverse the injection system.

## 6. Conclusion

A diagnostic-accessible ALD reactor capable of depositing device quality oxides using thermal ALD while being small enough to be easily integrated into an optical train was described. The operation of the reactor was demonstrated by measuring the time-dependent concentration profiles of TDMAT and water, two prototypical ALD precursors with significantly different mass diffusivity values. For both precursors, in a typical pressure and flow regime for ALD, purge times could be estimated using a simple exponential decay model with an exponential decay constant equal to the ratio between volumetric flow rate of the purge gas and the cell volume.
